# Supplementary material for: Effects of iron deficiency and exogenous sucrose on the intermediates of chlorophyll biosynthesis in Malus halliana
Source: PLoS One. 2020 May 6;15(5):e0232694. doi: 10.1371/journal.pone.0232694 (PMC7202898; doi:10.1371/journal.pone.0232694)
Supplement: Attachment — Submitted filename: Response to Reviewers.docx [file pone.0232694.s002.docx]

Dear Editors and Reviewers,

Thank you very much for your kind work and for the reviewers’ comments concerning our manuscript entitled “Effects of Iron Deficiency and Exogenous Sucrose on the Intermediates of Chlorophyll Biosynthesis in *Malus halliana*”. Those comments are all valuable and very helpful for revising and improving our paper, as well as the important guiding significance to our researches. We have studied comments carefully and have made correction which we hope meet with approval. We would like to revise manuscript if there are any requirements later. Revised portion are marked in red in the paper. The main corrections in the paper and the responses to the editor and reviewers’ comments are as following:

**ACADEMIC EDITOR:**

1. Thank you for submitting your manuscript to PLOS ONE. I agree with the reviewers that English language needs to be checked thoroughly. Main claims of the paper are not properly placed in the context of previous literature. Authors did not treat the literature fairly. Introduction is too general.

Response: English language has been checked and modified by American Journal Experts (AJE)

To express well the main claims of paper, we have added more related literature and deleted some unnecessary literature in introduction parts. All changes have been marked in red in the manuscript.

Add (1) “The Chl biosynthesis pathway has many steps and involves various enzymes, and a blockade in one step will affect Chl biosynthesis and cause changes in leaf color[7].” in line 44-46.

(2) “Chl biosynthesis plays essential roles in photosynthesis and plant growth in response to environmental change[6].” in line 42-44.

(3) “The key regulatory sites of Chl synthesis are different for each crop under external stress.” in line 46-47.

(4) “A study of adzuki bean reported that the transformation of protoporphyrin IX (Proto IX) is blocked in Chl synthesis, causing etiolated seedlings[8]. ” in line 47-48.

(5) “Remarkably, Fe deficiency directly affected Chl synthesis[12].” in line 55-56.

(6) “Research in poplar revealed that Chl synthesis was inhibited under Fe-deficient conditions[13].” in line 56-57.

(7) “Spiller et al. attempted to study the effect of Fe on the Chl biosynthetic pathway, and the results indicated that Fe deficiency leads to the accumulation and excretion of intermediates in the tetrapyrrole biosynthetic pathway, particularly coproporphyrin[14].” in line 57-60.

(8) “Moreover, an investigation was initiated to locate possible sites where a deficiency of Fe might limit Chl synthesis of cowpea plants[2].” in line 60-61.

(9) “However, the responses of Chl biosynthesis to Fe deficiency stress and the key regulatory sites in *M. halliana* are still unknown.” in line 62-63.

(10) “However, little is known about how exogenous sucrose regulates the response of M. halliana to Fe deficiency through chlorophyll synthesis.” in line 71-73.

(11) “Studies of Chl biosynthesis have focused on various aspects[23, 24], whether biochemical[25, 26] or genetic[27, 28].” in line 74-75.

(12) “However, gaps remain in the knowledge of Chl biosynthesis and the related genes in apple rootstocks.” in line 75-76.

(13) “Therefore, it is important to elucidate the Chl biosynthetic molecular responses of *M. halliana* to Fe deficiency.” in line 76-77.

(14) “This study is original research work investigating the differences between two apple rootstocks under Fe deficiency at a molecular scale.” In line 80-81.

2. If you would like to make changes to your financial disclosure, please include your updated statement in your cover letter.

Response: We have updated statement about funding in my cover letter.

3. To enhance the reproducibility of your results, we recommend that if applicable you deposit your laboratory protocols in protocols.io, where a protocol can be assigned its own identifier (DOI) such that it can be cited independently in the future. For instructions see: http://journals.plos.org/plosone/s/submission-guidelines#loc-laboratory-protocols

Response: We have deposited laboratory protocols in protocols.io, and DOI is dx.doi.org/10.17504/protocols.io.beb6jare.

**Additional Editor Comments (if provided):**

1. Thank you for submitting your manuscript to PLOS ONE. I agree with the reviewers that English language needs to be checked thoroughly. Main claims of the paper are not properly placed in the context of previous literature. Authors did not treat the literature fairly. Introduction is too general.

Response: English language has been checked and modified by American Journal Experts (AJE)

To express well the main claims of paper, we have added more related literature and deleted some unnecessary literature in introduction parts. All changes have been marked in red in the manuscript.

Add (1) “The Chl biosynthesis pathway has many steps and involves various enzymes, and a blockade in one step will affect Chl biosynthesis and cause changes in leaf color[7].” in line 44-46.

(2) “Chl biosynthesis plays essential roles in photosynthesis and plant growth in response to environmental change[6].” in line 42-44.

(3) “The key regulatory sites of Chl synthesis are different for each crop under external stress.” in line 46-47.

(4) “A study of adzuki bean reported that the transformation of protoporphyrin IX (Proto IX) is blocked in Chl synthesis, causing etiolated seedlings[8]. ” in line 47-48.

(5) “Remarkably, Fe deficiency directly affected Chl synthesis[12].” in line 55-56.

(6) “Research in poplar revealed that Chl synthesis was inhibited under Fe-deficient conditions[13].” in line 56-57.

(7) “Spiller et al. attempted to study the effect of Fe on the Chl biosynthetic pathway, and the results indicated that Fe deficiency leads to the accumulation and excretion of intermediates in the tetrapyrrole biosynthetic pathway, particularly coproporphyrin[14].” in line 57-60.

(8) “Moreover, an investigation was initiated to locate possible sites where a deficiency of Fe might limit Chl synthesis of cowpea plants[2].” in line 60-61.

(9) “However, the responses of Chl biosynthesis to Fe deficiency stress and the key regulatory sites in *M. halliana* are still unknown.” in line 62-63.

(10) “However, little is known about how exogenous sucrose regulates the response of M. halliana to Fe deficiency through chlorophyll synthesis.” in line 71-73.

(11) “Studies of Chl biosynthesis have focused on various aspects[23, 24], whether biochemical[25, 26] or genetic[27, 28].” in line 74-75.

(12) “However, gaps remain in the knowledge of Chl biosynthesis and the related genes in apple rootstocks.” in line 75-76.

(13) “Therefore, it is important to elucidate the Chl biosynthetic molecular responses of *M. halliana* to Fe deficiency.” in line 76-77.

(14) “This study is original research work investigating the differences between two apple rootstocks under Fe deficiency at a molecular scale.” In line 80-81.

**Journal Requirements:**

1. Please ensure that your manuscript meets PLOS ONE's style requirements, including those for file naming. The PLOS ONE style templates can be found at http://www.plosone.org/attachments/PLOSOne_formatting_sample_main_body.pdf and http://www.plosone.org/attachments/PLOSOne_formatting_sample_title_authors_affiliations.pdf

Response: We have revised the manuscript style according to style requirements. If the manuscript is still inappropriate, don't hesitate to contact me.

2. We noticed you have some minor occurrence of overlapping text with the following previous publication(s), which needs to be addressed: <https://bmcplantbiol.biomedcentral.com/articles/10.1186/s12870-015-0699-7>. In your revision ensure you cite all your sources (including your own works), and quote or rephrase any duplicated text outside the methods section. Further consideration is dependent on these concerns being addressed.

Response: We have addressed all overlapping text and cited all works sources in the introduction and discussion sections.

3. In your Methods, please state the source of the seeds used in your study.

Response: We have stated the source of seeds and marked in red in line 85-86.

4. While revising your submission, please upload your figure files to the Preflight Analysis and Conversion Engine (PACE) digital diagnostic tool, https://pacev2.apexcovantage.com/. PACE helps ensure that figures meet PLOS requirements.

Response: My uploaded figures have passed the PACE checks, and the PACE report signifies that the figure files need no adjustment.

**Reviewer #1:**

1. First you need to mention the common name in Title. You can mention scientific name in Abstract or Introduction in Parenthesis. Each scientific name has “L.” at the end according to nomenclature therefore, revise it thoroughly.

Response: We have submitted the article to AJE for language editing, and the title is recommended not to be changed. Additionally, we have revised thoroughly scientific name in abstract and introduction in parenthesis.

2. All abbreviations must be mentioned in full form at its first place. Mention all these abbreviations in footnotes of the first page.

Response: All abbreviations have been mentioned in full form at its first place, and all abbreviations have been mentioned in footnotes of the first page.

3. In the last paragraph of Introduction you have described some results. You just need to mention the background of your work as well as the significance for scientific world. You should discuss results in discussion part.

Response: We have moved some results in the Introduction section to the discussion part. In the meantime, we have added the background of work and the significance for scientific world in the introduction parts (line 55-75).

4. Insert line numbers in the manuscript for better review process.

Response: We have inserted line numbers in the manuscript.

5. Meaning is not clear “pylm”….is it Palm??

Response: We have confirmed again from the literature and determined that it is pylm and not Palm.

6. “nonphotosynthetic must be replaced with non-photosynthetic”

Response: This manuscript was edited and revised by the AJE. nonphotosynthetic cannot be replaced by non-photosynthetic.

7. You have mentioned “Increased Suc accumulation is required for regulating Fe-deficiency responses in plants, with auxins acting downstream in transmitting the Fe-deficiency signal”. How could you explain the downstreaming??

Response: This sentence comes from the 22_nd_ reference to show that sucrose is required for regulating Fe-deficiency responses in plants (See 22_nd_ reference).

8. “Exogenous Suc treatments”. Must mention Sucrose in full form in heading or subheadings. Similarly “Determination of Chl Precursors” as Determination of Chlorophyll Precursors.

Response: Sucrose in full form in heading or subheadings has been mentioned, and “Determination of Chl Precursors” has been replaced by “Determination of Chlorophyll Precursors”.

9. Caption of Fig 3. shows treatment codes as T1, T2 etc…all the punctuation must be appropriate like T3: Fe 21 d. Revise all codes in figure captions.

Response: We have revised all treatments codes in Fig 3 according to the above example.

10. You have used various abbreviations in your manuscript. You should also include Pn for Photosynthesis.

Response: We have added Pn (Photosynthesis) to the Abbreviation section.

11. “Stay green” must be replaced with greenness.

Response: “Stay green” has been replaced with “greenness”.

12. You must have to mention your main findings/conclusions under separate heading. Also mention your major outcomes in more elaborative way.

Response: We have mentioned main findings under separate heading and mentioned major outcomes in Results, Discussion and Conclusion parts.

13. Arrange the references alphabetically.

Response: Based on the PLoS ONE's style requirements, references should be arranged in the order in which they are inserted. So, we have arranged the references as requirements.

14. For the figures, you need to mention the x axis once for A, C, E and combine them, then mention x-axis scale once for B, D and F and combine them. Move Y-axis to right side of three figures (B, D, F). Similarly, move Y-axis on the left side of three figures (A, C, E). Combine all the six figures into single figure and enhance the dpi up to 500 for better understanding. Follow this procedure for Fig 1, 2, 3 and 5.

Response: We have generated the figures as required.

15. You should include a graphical abstract for clear understanding of the readers. You may modify Fig. 6 into graphical abstract after appropriate incorporation.

Response: We have tried to modified Fig 6 into graphical abstract. See Fig 6 and the Fig 6 legend (line 232-243) for the changes. We are willing to continue to modify when there are any requirements later.

**Reviewer #2:**

1. In Introduction part:

Need to revise these sentence

Inhibition of any steps of Chl synthesis will cause reduced Chl content.

A study investigating the biochemical mechanism of Chl deficiency in pylm by examining Chl biosynthesis precursors in this mutant has shown that Chl biosynthesis is blocked in the mutant at the Chl a production step

Response: We have revised and modified these sentences and the changes have been marked in red in the text.

2. Material and Method

In the material and method procedure regarding the isolation of RNA and preparation of cDNA was not discussed briefly.

Response: We have briefly added the isolation of RNA and preparation of cDNA in the material and method part.

3. In discussion part:

Fe deficiency stress upset the Chl biosynthesis balance in *M. hupehensis*, the Chl biosynthesis of *M. halliana* instead wasn’t blocked after Fe deficiency, which might be.

Besides, research has suggested that PbPPO1 genes might be involved in core browning under

modified atmosphere storage in ‘Yali’ pears[43].

Response: We have revised again these sentences as required, and the manuscript has been edited and modified by AJE. Changes are marked in red in the paper.

4. Need to italic the technical name

And PPO gene expression level against Aspergillus tubingensis in table grapes was enhanced with trehalose.

Response: The technical name has been changed to italic and marked in red in the text.

5. Need to revised this sentence

In this study, the *PPOX* gene was researched about the expression differences between two

apple rootstocks under Fe deficiency.

Response: We have deleted this sentence and restated in line 254-255.

We have added and revised details in the article according to your suggestions. Thank you again for improving our paper.

Best Regards,

Yours Sincerely

Ai-xia Guo

Corresponding author:

Name: Yan-xiu WANG

E-mail: wangxy@gsau.edu.cn
