# Supplementary material for: Effects of iron deficiency and exogenous sucrose on the intermediates of chlorophyll biosynthesis in Malus halliana
Source: PLoS One. 2020 May 6;15(5):e0232694. doi: 10.1371/journal.pone.0232694 (PMC7202898; doi:10.1371/journal.pone.0232694)
Supplement: Attachment — Submitted filename: Response to Reviewers.docx [file pone.0232694.s003.docx]

Dear Editors and Reviewers,

Thank you very much for your kind work and for the reviewers’ comments concerning our manuscript entitled “Effects of iron deficiency and exogenous sucrose on the intermediates of chlorophyll biosynthesis in *Malus halliana*”. Those comments are all valuable and very helpful for revising and improving our paper, as well as the important guiding significance to our researches. We have studied comments carefully and have made correction which we hope meet with approval. Revised portion are marked in red in the paper. The main corrections in the paper and the responses to the editor and reviewers’ comments are as following:

**Reviewer #1**

1. Even you have revised your paper from an English Editor but you cannot mention this “Iron1” in Title. You can show corresponding author email with asterisk that will also show abbreviation list in footnotes which is usually followed in literature.

Response: Thank you for your comments. We have revised “Iron1” in Title part, and we have showed corresponding author email with asterisk. All changes have been marked in red in the manuscript. If the manuscript is still inappropriate, don't hesitate to contact me.

2. In the first revision I have mentioned this but I can’t find this in text. Each scientific name has “L.” at the end according to nomenclature therefore, revise it thoroughly.

Response: Thank you very much for your suggestion and patience. We have referred to the relevant literature (the 4th and 45th references in text). The scientific name of two apple rootstock in the text may not include "L.". Then we consulted the academic editor. Editor has indicated that the change that reviewer#1 suggested is not necessary. Combining relevant literature (the 4th and 45th references in text) and editorial reply, we decided not to review the scientific name in the full text of my manuscript. If you have any questions or concerns about my manuscript, please don't hesitate to reach out and let me know.

3. In the first revision I have mentioned this but I can still find some related mistakes in the text. “Exogenous Suc treatments”. Must mention Sucrose in full form in heading or subheadings. Similarly “Determination of Chl Precursors” as Determination of Chlorophyll Precursors.

For example:

“Effects of Fe deficiency on the Chl precursor contents of *M. halliana* and *M. hupehensis*”

“Expression of genes involved in Chl biosynthesis under Fe deficiency”

“Regulation of the Fe deficiency phenotype by exogenous Suc”

Response: Thank you very much for your suggestion and patience. We have revised related mistakes in heading or subheadings. All changes have been marked in red in lines 81, 104, 126, 141, 142, 163, 164, 181, 198, 217 and 218.

4. For better understanding of the readers, you can move graphical abstract right after Abstract or Introduction before Materials and Methods. You can also quote this figure if there is any text relevant to this in the main body.

Response: Thank you very much for your suggestions. We can move graphical abstract right after Abstract or Introduction before Materials and Methods, but I think it's more understandable for the authors and readers without moving the graphical abstract. Additionally, this figure is a schematic model of key genes involved in chlorophyll biosynthesis of apple rootstock during iron deficiency and sucrose application. We haven't found a consistent graphics yet. Therefore, we didn’t quote this figure in the main body.

We have revised some details in the article according to your suggestions. Thank you again for improving our paper.

Best Regards,

Yours Sincerely

Ai-xia Guo

Corresponding author:

Name: Yan-xiu WANG

E-mail: wangxy@gsau.edu.cn
